# The Bispidinone Derivative 3,7-Bis-[2-(*S*)-amino-3-(1*H*-indol-3-yl)-propionyl]-1,5-diphenyl-3,7-diazabicyclo[3.3.1]nonan-9-one Dihydrochloride Induces an Apoptosis-Mediated Cytotoxic Effect on Pancreatic Cancer Cells In Vitro

**DOI:** 10.3390/molecules24030524

**Published:** 2019-01-31

**Authors:** Melanie J. Predebon, Danielle R. Bond, Joshua Brzozowski, Helen Jankowski, Fiona Deane, Mark Tarleton, Aron A. Shaw, Adam McCluskey, Michael C. Bowyer, Judith Weidenhofer, Christopher J. Scarlett

**Affiliations:** 1 Pancreatic Cancer Research Group, School of Environmental and Life Sciences, The University of Newcastle, Ourimbah, NSW 2258, Australia; melanie.predebon@uon.edu.au (M.J.P.); danielle.bond@newcastle.edu.au (D.R.B.); mark.tarleton@uon.edu.au (M.T.); michael.bowyer@newcastle.edu.au (M.C.B.); 2 School of Biomedical Sciences and Pharmacy, The University of Newcastle, Callaghan, NSW 2308, Australia; joshua.brzozowski@uon.edu.au (J.B.); helen.jankowski@uon.edu.au (H.J.); judith.weidenhofer@newcastle.edu.au (J.W.); 3 Cancer Program, Hunter Medical Research Institute (HMRI), New Lambton, NSW 2305, Australia; 4 Chemistry, School of Environmental and Life Sciences, The University of Newcastle, Callaghan, NSW 2308, Australia; fiona.deane@newcastle.edu.au (F.D.); aron.shaw@uon.edu.au (A.A.S.); adam.mccluskey@newcastle.edu.au (A.M.)

**Keywords:** Pancreatic cancer, bispidinone, cytotoxic, in vitro, apoptosis, drug development

## Abstract

Pancreatic cancer (PC) is a complex, heterogeneous disease with a dismal prognosis. Current therapies have failed to improve survival outcomes, urging the need for discovery of novel targeted treatments. Bispidinone derivatives have yet to be investigated as cytotoxic agents against PC cells. The cytotoxic effect of four bispidinone derivatives (**BisP1**: 1,5-diphenyl-3,7-bis(2-hydroxyethyl)-3,7-diazabicyclo[3.3.1]nonan-9-one; **BisP2**: 3,7-bis-(2-(S)-amino-4-methylsulfanylbutyryl)-1,5-diphenyl-3,7-diazabicyclo[3.3.1]nonan-9-one dihydrochloride; **BisP3:** [2-{7-[2-(*S*)-*tert*-butoxycarbonylamino-3-(1*H*-indol-3-yl)-propionyl]-9-oxo-1,5-diphenyl-3,7-diazabicyclo[3.3.1]non-3-yl}-1-(*S*)-(1*H*-indol-3-ylmethyl)-2-oxoethyl]-carbamic acid tertbutyl ester; **BisP4**: 3,7-bis-[2-(*S*)-amino-3-(1*H*-indol-3-yl)-propionyl]-1,5-diphenyl-3,7-diazabicyclo[3.3.1]nonan-9-one dihydrochloride) was assessed against PC cell lines (MiaPaca-2, CFPAC-1 and BxPC-3). Cell viability was assessed using a Cell Counting Kit-8 (CCK-8) colorimetric assay, while apoptotic cell death was confirmed using fluorescence microscopy and flow cytometry. Initial viability screening revealed significant cytotoxic activity from **BisP4** treatment (1 µM–100 µM) on all three cell lines, with IC_50_ values for MiaPaca-2, BxPC-3, and CFPAC-1 16.9 µM, 23.7 µM, and 36.3 µM, respectively. Cytotoxic treatment time-response (4 h, 24 h, and 48 h) revealed a 24 h treatment time was sufficient to produce a cytotoxic effect on all cell lines. Light microscopy evaluation (DAPI staining) of **BisP4** treated MiaPaca-2 PC cells revealed dose-dependent characteristic apoptotic morphological changes. In addition, flow cytometry confirmed **BisP4** induced apoptotic cell death induction of activated caspase-3/-7. The bispidinone derivative **BisP4** induced an apoptosis-mediated cytotoxic effect on MiaPaca-2 cell lines and significant cytotoxicity on CFPAC-1 and BxPC-3 cell lines. Further investigations into the precise cellular mechanisms of action of this class of compounds are necessary for potential development into pre-clinical trials.

## 1. Introduction

Pancreatic cancer (PC) remains a devastating disease; it is the fourth most common cause of cancer death in the Western world [1] and is projected to be the second leading cause of cancer death by 2030 [2]. Newly diagnosed PC patients have a median survival of six months and a five-year survival rate of less than 7% [1]. PC is an inherently metastatic disease, limiting the effectiveness of local therapies, such as surgery and radiation [3]. A major challenge of PC is that it is highly resistant to current chemotherapy drugs [4] and without effective chemotherapy options available for PC patients, a pressing need exists for the continued discovery of novel drug candidates. 

Drug development from synthetic compounds and synthetic analogues of pharmacophores in natural products provides the benefit of structural modification of drug candidates to improve potency, selectivity, and bioavailability [5,6]. Plant alkaloids in particular are traditional starting points for drug development, with many compounds in this class possessing potent biological activity. The bispidine ring system (3,7-diazabicyclo[3.3.1]nonane) is an unusual heterocyclic compound consisting of two piperidine rings fused at a common three carbon junction and is the base structure for the quinolizidine plant alkaloids, including sparteine and related analogues cytisine and anagyrine. Sparteine exhibits antiarrhythmic and antibacterial properties, while cytisine and associated derivatives display analgesic, antihypertensive, antispasmodic, and antidepressant activities [7,8,9,10,11]. 

The ability of the bispidine ring to adopt different conformational forms in solution (i.e., chair-chair, chair-boat, boat-boat) has been a point of significant research interest, prompting a number of spectroscopic studies [12,13]. This behavior in turn gives rise to the possibility of exo/endo isomeric forms of substituents hosted on the methylene C2, C4, C6, and C8 ring positions leading to further structural diversity. These features, coupled with the high basicity of the ring nitrogen atoms (N3 and N7), make the bispidine system an interesting choice as a privileged scaffold for drug development. A recent review article by Tomassoli and Gundisch provides an excellent overview of the physical, chemical, and pharmacological history of the bispidine system together with an overview of physical, chemical, and spectroscopic properties [14]. 

Bispidine moieties have been investigated as potential scaffolds for inducing secondary structures in peptides, selected because of their rigidity and lipophilic surface area, potentially enabling interaction with cellular/viral proteins and cell membranes [15]. Bispidine-amino acid conjugates have also been investigated as novel scaffolds for antiviral applications [16]. 

Bispidinones—3,7-diazabicyclo[3.3.1]nonan-9-ones (3,7-DABN)—are a synthetic bispidine derivatives, distinguished by incorporation of a carbonyl unit at C9. Symmetric *N*,*N*′-substituted bispidinones can be prepared using a simple one-pot Mannich reaction [17,18], while asymmetric *N*,*N*′-compounds are generated via a step-wise approach [19,20]. The number, type, and position of functional groups can be varied during bispidinone synthesis, providing a variety of tailor-made potential bioactive molecules [21]. 

**Figure 1:**
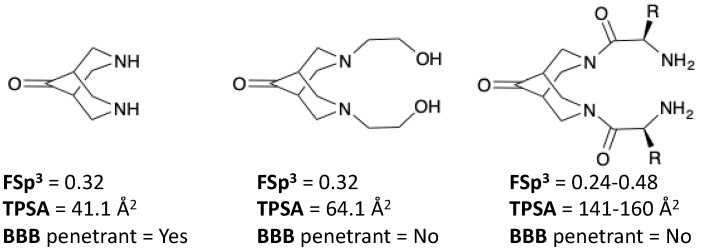
SwissADME (http://www.swissadme.ch) predicted properties of a selected series of bispidinone analogues. The introduction of N-substituents facilitated modulation of the fraction of sp3 hybridized carbon atoms (Fsp^3^) character, increased topological polar surface area (TPSA), and predicted no blood brain barrier (BBB) penetration.

In contrast to bispidines, few studies investigating the biological properties of bispidinone derivatives and their potential biological effects, in particular regarding anti-cancer activity have to date been undertaken [22,23]. In these cases, considerable variation in substituent attachment and pattern exists making further study of the system essential to develop a more comprehensive understanding of structure-activity relationships. This present study is the first to assess whether selected bispidinone compounds exert cytotoxic effects on pancreatic cancer cell lines. In this initial study we specifically targeted analogues predicted to be blood brain barrier (BBB) impenetrable, readily synthesizable, and allow rapid and subtle modification of analogue physicochemical properties, e.g., topological polar surface area (TPSA) and the fraction of sp3 hybridized carbon atoms (Fsp^3^) (Figure 1).

## 2. Results

### 2.1. Synthesis of BisP1-BisP4

**Scheme 1:**
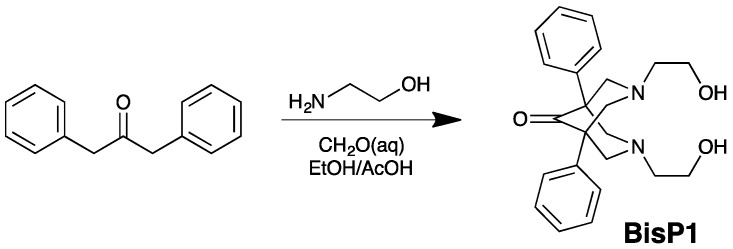
Synthesis of 1,5-diphenyl-3,7-bis(2-hydroxyethyl)-3,7-diazabicyclo[3.3.1]nonan-9-one (**BisP1**).

The desired symmetrical bispidinones were prepared from readily accessible starting materials. **BisP1** was accessed via a “twin double Mannich reaction” from 1,3-diphenylacetone, ethanolamine, and aqueous formaldehyde (Scheme 1) [24].

**Scheme 2:**
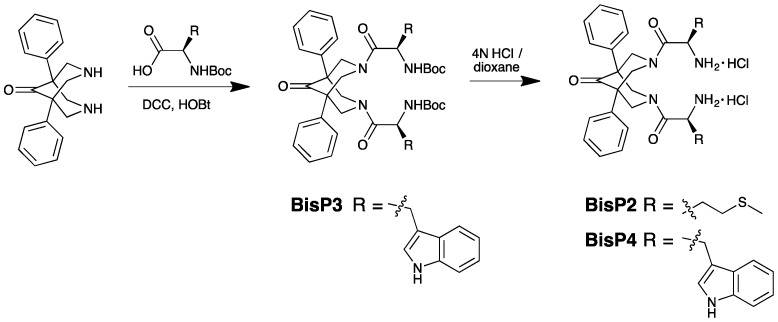
Synthesis of **BisP2**, **BisP3**, and **BisP4**.

The bispidinones **BisP2**–**BisP4** were prepared from the easily accessed 1,5-diphenyl-3,7-diazabicyclo[3.3.1]nonan-9-one, through an amide bond formation reaction employing HOBt and DCC as coupling reagents (Scheme 2). In all cases, substitution occurred at both aza moieties and products were obtained in high yields (73–93%). The configuration of the amide bonds in these bispidinone species results in the formation of two distinct conformational isomers: a *C*_2_ symmetric species wherein the amide moieties are aligned in an anti-parallel orientation and a *C*_1_ symmetric isomer with the amide groups in a parallel orientation. The anti-parallel configuration is more energetically favorable due to the lack of steric interaction between the amide substituents. Removal of the Boc protecting group was achieved using 4N HCl in dioxane at room temperature to afford the desired amine salts as solids in high yields.

### 2.2. Cytotoxic Effect of Bispidinone Treatment

**Figure 2:**
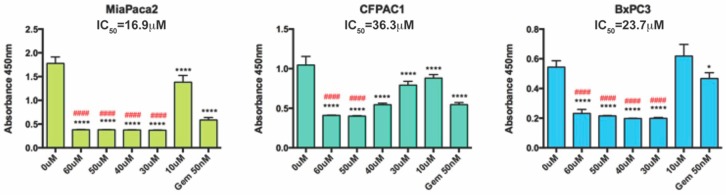
After 48 h treatment, derivative **BisP4** had a statistically significant reduction in cell viability from all concentrations, except for 10 µM treatment on BxPC3 cells. Results are mean absorbance ± SD, n = 6. Values for variance (*p*) between control versus treatment and gemcitabine versus treatment are respectively denoted by symbols ****,^#^^###^, *p* < 0.0001, ***,^###^ *p* < 0.001, **,^##^ *p* < 0.01, *,^#^ *p* < 0.05, determined using a one-way ANOVA and Dunnett’s post-hoc test.

The cytotoxicity of the bispidinone derivatives was determined using Cell Counting Kit-8 (CCK-8) colorimetric assays after 48 h of treatment. Results from the initial screening of the four bispidinone derivatives (**BisP1**, **BisP2**, **BisP4**, and **BisP3**) on three pancreatic cancer cell lines (BxPc-3, CFPAC-1, and MiaPaca-2) revealed that derivative **BisP4** was the most effective in reducing cell viability. The parent 1,5-diphenyl-3,7-diazabicyclo[3.3.1]nonan-9-one was not examined in our assays as it failed our preliminary in silico analysis displaying predicted BBB penetration. **BisP4** showed significant cytotoxic activity (<50 µM) when compared to the control (Table 1). In contrast, the bispidinone derivative **BisP1** demonstrated no significant cytotoxic effects and bispidinone **BisP2** was cytotoxic to the pancreatic cancer cell lines at doses of 100 µM and above (Table 1), which was determined to be too high to be considered therapeutically relevant [25] and was therefore excluded from further investigation. IC_50_ values were then calculated to compare **BisP4** treatment between pancreatic cell lines. **BisP4** had the lowest IC_50_ value for MiaPaca-2 cells (16.9 µM), followed by BxPC-3 (23.7 µM) and CFPAC-1 (36.3 µM) (Figure 2). Consequently, MiaPaca-2 cells were selected for continued investigation into the mechanisms of action of the cytotoxic effects of **BisP4**.

### 2.3. **BisP4** Induces Apoptosis in Pancreatic Cancer Cells 

**Figure 3:**
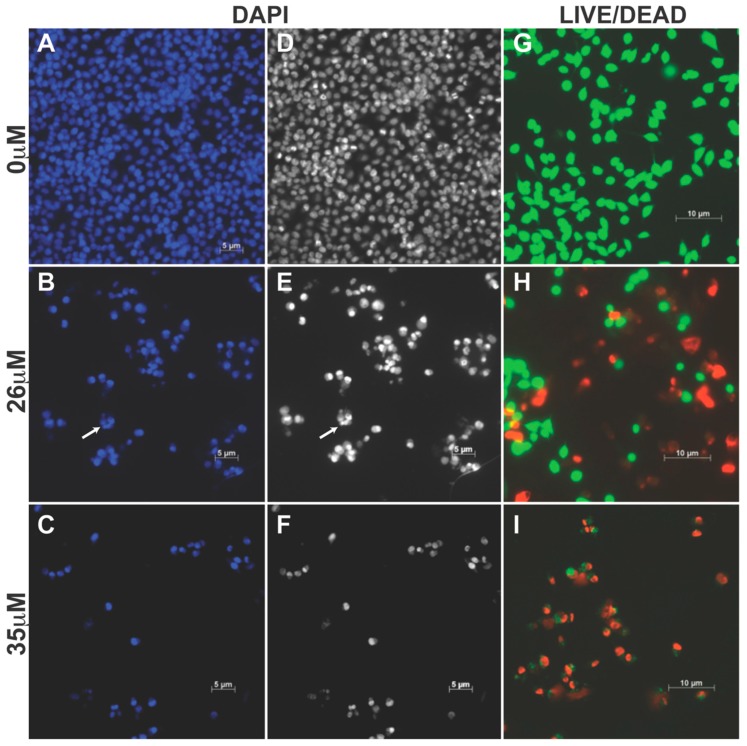
Characteristic apoptotic morphological changes in MiaPaca-2 cells after 24 h treatment with **BisP4**: (**A**,**D**,**G**) 0 µM; (**B**,**E**,**H**) 26 µM (**C**,**F**,**I**) 35 µM. The DAPI stain (**A**–**F**) revealed morphological changes indicative of apoptosis in a dose-dependent manner. BisP4 treated MiaPaca-2 cells display distinct apoptotic bodies (arrow), as a result of increased DAPI staining of fragmented nuclear bodies. At the higher concentration (35 µM) the cells show an intense fluorescence corresponding to chromatin condensation and fragmentation, synonymous with apoptosis. (**G**–**I**): Decreased live cells (green) and increased dead cells (red) following treatment with increasing doses of BisP4 (26 µM and 35 µM). The compromised plasma membrane integrity of the dead or dying cells allows uptake of ethidium homodimer-1 (EthD-1), which fluoresces red.

Morphological changes were observed indicative of apoptosis in a dose-dependent manner on the **BisP4** treated MiaPaca-2 cells. MiaPaca-2 cells treated with vehicle (Figure 3A,D) showed no indication of apoptosis with a homogenous weak blue stain throughout the cell. In contrast, **BisP4** treated MiaPaca-2 cells (Figure 3B,E) displayed strong punctate DAPI indicative of fragmented nuclear bodies. Further, a higher dose treatment of MiaPaca-2 cells with **BisP4** resulted in more intense fluorescence consistent with chromatin condensation and fragmentation, synonymous with apoptosis (Figure 3C,F). In addition, the fluorescence micrographs show a decrease in cell numbers in a dose-dependent manner. 

The LIVE/DEAD assay was applied to the **BisP4** treated and control cells, which revealed significant compromised cell membrane changes of MiaPaca-2 cells in a dose-dependent manner. No dead cells were identified in the vehicle treatment as determined by the absence of red fluorescence, whereas the proportion of dead cells increased with the dose of **BisP4** (Figure 3G,H,I).

### 2.4. Cell Death Analysis by Flow Cytometry

**Figure 4:**
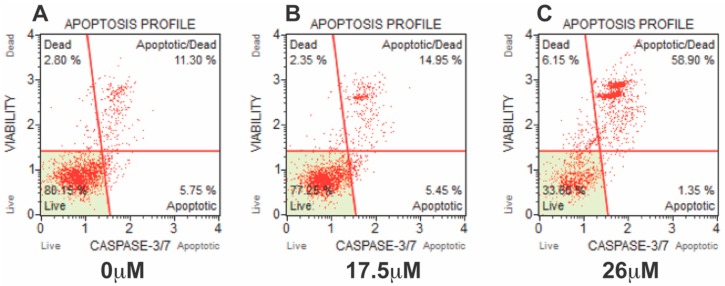
Caspase 3/7 analysis to assess apoptosis of MiaPaca-2 cells after 24h treatment with BisP4: (**A**) 0 µM (no compound); (**B**) 17.5 µM; and (**C**) 26 µM. The lower left quadrant depicts live cells not undergoing detectable apoptosis; the lower right quadrant shows cells in the early stages of apoptosis; the upper right quadrant shows cells in the late stage of apoptosis or dead by apoptotic mechanisms; and the upper left quadrant shows cells that died via necrosis but not through the apoptotic pathway.

To further evaluate how **BisP4** induces apoptosis on MiaPaca-2 cells, a caspase-3/-7 flow cytometry assay was used. **BisP4** treatment (17.5 µM and 26 µM) induced a dose-dependent increase in the proportion of apoptotic MiaPaca-2 cells (Figure 4 and Table 2), with negligible effects on necrosis.

## 3. Discussion

Treatment options for pancreatic cancer patients remain limited and over the last 30 years there has yet to be any significant advances from clinical drug trials that improve patient survival [26,27]. Due to the known complexity and heterogeneity of pancreatic cancer [28,29,30], there are potentially significant benefits in the continued discovery of novel targeted drug treatments. Recent research has shown that tetraaryl functionalised bispidinone analogues induce apoptosis in HeLa cervical cancer cell lines [22,23]. However, no studies to date have assessed the potential effects of aliphatic and aromatic *N*,*N*-disubstituted bispidinone derivatives on PC cell lines. 

This study investigated the anti-pancreatic cancer activity of four rationally synthesized bispidinone derivatives (**BisP1**, **BisP2**, **BisP4**, and **BisP3**). The bispidinone 3,7-bis-[2-(*S*)-amino-3-(1*H*-indol-3-yl)-propionyl]-1,5-diphenyl-3,7-diazabicyclo[3.3.1]nonan-9-one dihydrochloride (**BisP4**), containing tryptophan pendant arm substituents, was identified to be the most effective of the derivatives evaluated in reducing viability of PC cells. **BisP4** showed statistically significant cytotoxic activity, with MiaPaca-2 cells being the most sensitive, showing significant activity at concentrations well below 20 µM.

Following the observed cytotoxic activity of derivative **BisP4**, it was determined that comparison activity should be undertaken against the almost structurally identical Boc-protected derivative, labelled **BisP3.** The Boc (*tert*-butyloxycarbonyl) group present on **BisP3** serves as a protection group during synthesis, preventing chemical transformation of the α-amino group of tryptophan [31]. The goal of screening **BisP3** and comparing cell viability to **BisP4** was to examine the structure activity relationship on the cytotoxic effect. These data also highlight the benefit of synthetic drug design, in that structural modification can vary steric and electronic properties to improve drug action and/or efficacy. Derivative **BisP3** was screened and cell viability compared to the de-protected **BisP4** using the same treatment concentrations, however **BisP3** had poor solubility in the culture media, often forming a precipitate. This degree of insolubility is likely to explain the lack of cytotoxic activity shown in the results compared to **BisP4** treatment at similar concentrations. 

To further support the viability results for **BisP4**, an additional fluorescent cytotoxicity assay was performed. MiaPaca-2 cells were treated with a range of concentrations of **BisP4** with increasing numbers of dead cells, observed in a dose-dependent manner. 

Indicators of apoptotic cell death followed **BisP4** treatment were observed, such as morphological and biochemical changes like caspase-3/-7 activation. Defects in cell death pathways are one of the defining hallmarks of cancer [32] and many current chemotherapy drugs kill cancer cells by triggering apoptosis [33,34]. Thus, this study investigated the presence of apoptotic markers to determine if **BisP4** induced PC cell death was a result of apoptosis. Following a 24 h treatment of **BisP4** on MiaPaca-2 cells clear apoptotic characteristics, i.e. nuclear segmentation, apoptotic body formation, and chromatin condensation, occurred in a dose-dependent manner. 

Overall, the results revealed apoptotic morphology following **BisP4** treatment, which increased in a dose-dependent manner. These results agree with two recent studies investigating the cytotoxic effect of bispidinone analogues on HeLa cervical cancer cells in vitro [22,23]. Both studies reported significant apoptosis-mediated cytotoxicity from two bispidinone analogues: 2,4,6,8-tetrakis(2-methoxyphenyl)-3,7-diazabicyclo[3.3.1]nonan-9-one [22] and 2,4,6,8-(3)-tetranitrophenyl-3,7-diazabicyclo[3.3.1]nonan-9-one [23]. Together, these results emphasize how apoptotic events can have a different time course depending on the concentration of the cytotoxic agent.

Apoptosis is also characterized by activating the caspase cascade that ends in the executioner caspases-3 and -7, which results in cleavage of numerous structural and signaling proteins [33]. The caspase-3/-7 assay revealed a decrease in the percentage of live cells following **BisP4** treatment in a dose-dependent manner. These results further support our findings that the cytotoxic effect of derivative **BisP4** on MiaPaca-2 cells can be attributed to apoptosis. 

While the results presented in this study are preliminary in nature, a number of conclusions can be draw from the findings. All compounds assessed possess a common bispidinone scaffold and are *C*_2_ symmetric in form. Bispidinones possessing short chain aliphatic pendant arms (**BisP1** = O, **BisP2** = S) exhibit no significant biological activity at concentrations <50 μM. Inclusion of pendant arms containing electron rich character and hydrogen bond donor/acceptor capability induces a dramatic change in cellular toxicity in compound **BisP4**. All compounds assessed (**BisP2**–**BisP4**) contain amide units at the N3 and N7 ring positions that introduce sp2 character to the pendant arm, yet only **BisP4** displays significant biological activity suggesting that cytotoxic activity may be derived from the electronic characteristics present in the amino acid residue. 

## 4. Materials and Methods 

### 4.1. Chemistry General Procedures

All reactions were performed using standard laboratory equipment and laboratory glassware. Solvents and reagents were purchased from Sigma-Aldrich (St Louis, MO, USA), Matrix Scientific (Columbia, SC, USA), or Lancaster Synthesis (Ward Hill, MA, USA) and were used as received. Organic solvents were of bulk quality and re-distilled from glass prior to use. Flash chromatography was conducted using silica gel 200–400 mesh (60 Å). Organic solvent extracts were dried over MgSO_4_ and removed in vacuo with either a Büchi (Flawil, Switzerland) or Heidolph (Schwabach, Germany) rotary evaporator. Melting points were recorded in open capillaries on a Brüker 565 Melting Point Apparatus (Brüker, Billerica, MA, USA), with temperatures expressed in degrees Celsius (°C). Infrared spectra were recorded on a Shimadzu FTIR-8400 (Shimadzu, Kyoto, Japan), from 4000 cm^−1^ to 800 cm^−1^. Organic solids and oils were measured as either thin film anhydrous CHCl_3_ solutions between sodium chloride plates or as a cast on a sodium chloride plate.

Concerning compounds **BisP1** and **BisP3**, electron impact mass spectra were determined on a Shimadzu GCMS-QP5050A instrument, using the DI probe and operating conditions of 70 eV and 8000 V accelerating potential. A 20 min temperature program, starting at 100 °C and increasing by 20 °C min^−1^ up to 400 °C, was followed. Electrospray Ionisation mass spectra were measured on a VG Platform II single quadrupole mass spectrometer coupled to a HPLC binary pump system (Agilent, Santa Clara, CA, USA). The capillary tip was at a potential of ±3.5 kV relative to ground, the source temperature was maintained at 80 °C, and the cone voltage was varied between +20 to +60 for positive-ion and −20 to −50 V for negative ion mode. The electrospray mass spectra of **BisP2** and **BisP4** were recorded using 10% DMSO/H_2_O or HPLC-grade methanol or acetonitrile as carrier solvents on an Agilent 1260 LCMS system). The mass spectral data is reported according to the following scheme: ion peak (*m*/*z*) and intensity. The molecular ion is denoted by (M^+^).

^1^H NMR spectra were recorded at 300 MHz using a Brüker Avance DPX-300 spectrometer or a Brüker Avance III 400 MHz spectrometer. ^13^C NMR spectra were recorded at 75 MHz using a Brüker Avance DPX-300 spectrometer or 100 MHz on a Brüker Avance III 400 MHz. All spectra were recorded in deuterated dimethyl sulfoxide (DMSO-*d_6_*) or deuterated chloroform (CDCl_3_) with 0.1% *v/v* tetramethylsilane (TMS) as an internal standard (δ 0.0 parts per million (ppm)), obtained from Sigma Aldrich or Cambridge Isotope Laboratories Inc. (Tewkesbury, MA, USA). The residual protonated solvent peaks were used as the internal reference (δ 2.49 (quintet) and δ 39.7 (septet) for ^1^H NMR and ^13^C NMR respectively). Chemical shifts (δ) were measured in parts per million (ppm) and referenced against the internal reference peaks. Coupling constants (*J*) were measured in Hertz (Hz). Multiplicities are denoted as singlet (s), broad singlet (br s), doublet (d), doublet of doublets (dd), doublet of doublet of doublets (ddd), triplet (t), triplet of doublets (td), doublet of triplets (dt), quartet (q), quintet (quin), and multiplet (m). Peaks are listed in increasing chemical shift in the following format: chemical shift (integration (^1^H), multiplicity (^1^H), and coupling constant (^1^H)).

### 4.2. Preparation of Bispidinone Compounds

Four bispidinone derivatives were synthesized (Table 1) as follows:

**BisP1:** 1,5-Diphenyl-3,7-bis(2-hydroxyethyl)-3,7-diazabicyclo[3.3.1]nonan-9-one

**BisP2:** 3,7-Bis-(2-(*S*)-amino-4-methylsulfanylbutyryl)-1,5-diphenyl-3,7-diazabicyclo[3.3.1]nonan-9-one dihydrochloride

**BisP3:** [2-{7-[2-(*S*)-*tert*-Butoxycarbonylamino-3-(1*H*-indol-3-yl)-propionyl]-9-oxo-1,5-diphenyl-3,7-diazabicyclo[3.3.1]non-3-yl}-1-(*S*)-(1*H*-indol-3-ylmethyl)-2-oxoethyl]-carbamic acid *tert*-butyl ester 

**BisP4**: 3,7-Bis-[2-(*S*)-amino-3-(1*H*-indol-3-yl)-propionyl]-1,5-diphenyl-3,7-diazabicyclo[3.3.1]nonan-9-one dihydrochloride

**1,5-Diphenyl-3,7-bis(2-hydroxyethyl)-3,7-diazabicyclo[3.3.1]nonan-9-one (BisP1)**

To a mixture of 1,3-diphenylacetone (2.1 g, 0.01 mol) and ethanol (20 mL) was added ethanolamine (1.20 mL, 0.02 mol) and aqueous formaldehyde (3.24 mL, 0.04 mol). The resultant mixture was heated under reflux overnight. After this time, the reaction mixture was chilled and the resulting white precipitate collected and washed with diethylether to afford **BisP1**. Yield: 3.54 g, 93%. m.p. 182–184 °C. IR (cm^−1^): 3465, 3056, 2945, 2825, 1721, 1600, 1494, 1445, 1344, 1296, 1272, 1208, 1183, 1117, 1080, 1039, 933, 836. 

^1^H NMR (CDCl_3_) (300 MHz): δ 2.73–2.77 (t, *J* = 5.0 Hz, 4H, exocyclic NCH_2_), 3.19–3.27 (d, *J* = 11.5 Hz, 4H, ring CH_2_), 3.63–3.71 (d, *J* = 11.5 Hz, 4H, ring CH_2_), 3.74–3.78 (t, *J* = 5.0 Hz, 4H, CH_2_OH), 4.94 (br, 2H, OH), 7.30–7.41 (m, 10H, aryl).

^13^C NMR (CDCl_3_) (75 MHz): δ 54.7 (CPh), 58.3 (CH_2_OH), 58.6 (exocyclic NCH_2_), 65.3 (ring CH_2_), 127.2 (aryl), 127.3 (aryl), 128.0 (aryl), 138.1 (aryl), 209.8 (CO).

Mass Spectrum (EI): *m*/*z* 380 (11%) M^+^, 362 (17), 307 (45), 292 (86), 276 (19), 248 (13), 234 (42), 204 (8), 190 (27), 176 (24), 144 (14), 115 (57), 103 (76), 91 (68), 88 (100), 77 (33), 72 (36), 58 (70), 44 (66), 42 (58). LRMS (ESI M + 1): 381

HRMS (ESI M + H) for C_23_H_29_N_2_O_3_: calculated 381.2173, found 381.2171

**3,7-Bis-(2-(*S*)-amino-4-methylsulfanylbutyryl)-1,5-diphenyl-3,7-diazabicyclo[3.3.1]nonan-9-one dihydrochloride (BisP2)**

To a rapidly stirred mixture of 1,5-diphenyl-3,7-diazabicyclo[3.3.1]nonan-9-one (0.31 g, 1.06 mmol), *N*-(*tert*-butoxycarbonyl)-l-methionine (0.53 g, 2.13 mmol) and 1-hydroxy benzotriazole (HOBt) hydrate (0.29 g, 2.15 mmol) in CH_2_Cl_2_ (30 mL), was added a solution of 1,3-dicyclohexylcarbodiimide (DCC) (0.44 g, 2.14 mmol) in CH_2_Cl_2_ (20 mL). The mixture was stirred overnight and cooled to 4 °C. The precipitate was collected and washed with citric acid (10% *v*/*v*, 2 × 100 mL), saturated sodium bicarbonate (2 × 100 mL), and brine (100 mL). The organic layer was dried (MgSO_4_) and evaporated in vacuo. The residue was purified by flash chromatography (EtOAc:CH_2_Cl_2_ 1:20) to afford {1-(*S*)-[7-(2-(*S*)-*tert*-butoxycarbonylamino-4-methylsulfanylbutyryl)-9-oxo-1,5-diphenyl-3,7-diazabicyclo[3.3.1]nonane-3-carbonyl]-3-methylsulfanylpropyl}-carbamic acid tert-butyl ester (Yield: 0.62 g, 77%).

A 4M HCl solution in dioxane (5 mL) was added to the freshly prepared {1-(*S*)-[7-(2-(*S*)-*tert*-butoxycarbonylamino-4-methylsulfanylbutyryl)-9-oxo-1,5-diphenyl-3,7-diazabicyclo[3.3.1]nonane-3-carbonyl]-3-methylsulfanylpropyl}-carbamic acid *tert*-butyl ester (0.20 g, 0.268 mmol) from above and stirred at room temperature overnight. The mixture was evaporated in vacuo to dryness and cold ether (5 mL) was added. The resulting precipitate was collected and dried under high vacuum (0.01 mm Hg) for 48 h to afford **BisP2** as an off-white solid (0.16 g, 95%). m.p. 199 °C decomp., IR (cm^−1^): 3334, 2927, 2852, 1731, 1634, 1653, 1627, 1635, 1559, 1490, 1379, 1296, 1257, 1054, 760, 698, 617, 510. 

^1^H NMR (CDCl_3_) (300 MHz): δ 1.89–1.96 (m, 2H, CHCH_2_CH_2_S), 2.11 (s, 6H, SCH_3_), 2.41–2.51 (m, 4H, CH_2_S), 3.42–3.52 (d, *J* = 13.7 Hz, 2H, ring CH_2_), 3.87–3.96 (d, *J* = 13.5 Hz, 2H, ring CH_2_), 4.02–4.06 (m, 2H, (C(O)CH), 4.62–4.71 (d, *J* = 13.5 Hz, 2H, ring CH_2_), 5.47–5.57 (d, *J* = 13.7 Hz, 2H, ring CH_2_), 7.19 –7.44 (m, 10H, aryl), 8.55 (s, 6H, NH_3_^+^).

^13^C NMR (CDCl_3_) (75 MHz): δ 18.3 (SCH_3_), 31.0 (CH_2_S), 35.7 (CHCH_2_CH_2_S), 50.6 (C(O)CH), 52.6 (ring CH_2_), 53.5 (CPh), 56.4 (ring CH_2_), 127.7 (aryl), 128.5 (aryl), 128.7 (aryl), 135.9 (aryl), 173.3 (NC(O)CH), 207.2 (CO).

Mass Spectrum (ES^−^): *m*/*z* 555 (M−H)^−^ (100%), 157 (35).

LRMS (ESI M + 1): 555

HRMS (ESI M + H) for C_29_H_39_N_4_O_3_S_2_: calculated 555.2458, found 555.2468

**[2-{7-[2-(*S*)-*tert*-Butoxycarbonylamino-3-(1*H*-indol-3-yl)-propionyl]-9-oxo-1,5-diphenyl-3,7-diazabicyclo[3.3.1]non-3-yl}-1-(*S*)-(1*H*-indol-3-ylmethyl)-2-oxoethyl]-carbamic acid *tert*-butyl ester (BisP3)**

To a rapidly stirred mixture of 1,5-diphenyl-3,7-diazabicyclo[3.3.1]nonan-9-one (0.31 g, 1.06 mmol), *N*-(*tert*-butoxycarbonyl)-l-tryptophan (0.63 g, 2.07 mmol), and 1-hydroxybenzotriazole (HOBt) hydrate (0.28 g, 2.07 mmol) in CH_2_Cl_2_ (30 mL), was added a solution of 1,3-dicyclohexylcarbodiimide (DCC) (0.43 g, 2.09 mmol) in CH_2_Cl_2_ (20 mL). The mixture was stirred overnight, cooled to 4 °C, where the precipitate collected and washed with citric acid (10% *v*/*v*, 2 × 100 mL), saturated sodium bicarbonate (2 × 100 mL), and brine (100 mL). The organic layer was dried (MgSO_4_) and evaporated in vacuo. The residue was recrystallized from ethanol to give **BisP3** as fine white crystals. Yield: 0.79 g, 86%. m.p. 206–208°C. IR (cm^−1^): 3418, 3392, 3026, 2993, 2946, 2871, 1728, 1709, 1663, 1601, 1583, 1505, 1436, 1379, 1230, 1172, 1105, 1079, 1031, 824. 

^1^H NMR (CDCl_3_) (300 MHz): δ 1.60 (s, 18H, *boc*CH_3_), 1.95–2.03 (d, *J* = 13.3 Hz, 2H, ring CH_2_), 2.64–2.74 (d, *J* = 13.9 Hz, 2H, ring CH_2_), 2.87–2.95 (t, *J* = 13.4 Hz, 2H, CH_2_-indole), 3.25–3.35 (t, *J* = 13.6 Hz, 2H, CH_2_-indole), 3.85–3.93 (d, *J* = 13.3 Hz, 2H, ring CH_2_), 4.89–4.99 (d, *J* = 13.9 Hz, 2H, ring CH_2_), 5.10–5.14 (m, 2H, CONHCH), 5.95 (br, 2H, NH), 6.22–6.26 (d, *J* = 7.4 Hz, 4H, indole-H), 7.13–7.30 (m, 14H, indole-H and aryl), 7.68–7.74 (d, *J* = 7.7 Hz, 2H, indole-H), 8.07 (s, 2H, indole-NH).

^13^C NMR (CDCl_3_) (75 MHz): δ 29.5 (*boc*CH_3_), 32.7 (CH_2_-indole), 51.5 CONHCH), 53.1 (ring CH_2_), 53.7 (CPh), 55.7 (ring CH_2_), 80.5 (C(CH_3_)), 112.0 (indole CH), 119.9 (indole CH), 120.8 (indole CH), 123.1 (indole CH), 127.5 (aryl), 128.3 (indole CH), 128.8 (aryl), 129.0 (aryl), 135.1 (indole-C), 136.7 (aryl), 156.2 (NC(O)O), 172.0 (NC(O)CH), 207.6 (CO).

Mass Spectrum (EI): *m*/*z* 864 (1%) M^+^, 764 (3), 734 (1), 677 (1), 633 (1), 604 (2), 577 (6), 563 (1), 535 (3), 478 (9), 447 (1), 291 (2), 261 (2), 248 (18), 234 (7), 205 (1), 185 (6),144 (5), 132 (6), 130 (3), 116 (24), 103 (13), 101 (100), 91 (38), 77 (8), 57 (63), 44 (51).

LRMS (ESI M + 1): 865

HRMS (ESI M + H) for C_51_H_57_N_6_O_7_: calculated 865.4283, found 865.4289

**3,7-Bis-[2-(*S*)-amino-3-(1*H*-indol-3-yl)-propionyl]-1,5-diphenyl-3,7-diazabicyclo[3.3.1]nonan-9-one dihydrochloride (BisP4)**

To a vial containing **BisP3** (0.207 g, 0.248 mmol) was added a solution of 4M HCl in dioxane (5 mL); the reaction mixture was stirred at room temperature overnight. After this time, the reaction mixture was evaporated under reduced pressure and cold diethyl ether (5 mL) added. The resulting precipitate was collected and dried under high vacuum (0.01 mm Hg) for 48 h to afford **BisP4** as an off-white solid with a yield of 93% (0.18 g) m.p. 230 °C decomp., IR (cm^−1^): 3404, 3328, 3185, 3055, 2982, 2929, 1731, 1653, 1490, 1459, 1447, 1378, 1343, 1261, 1098, 746, 698. 

^1^H NMR (CDCl_3_) (300 MHz): δ 2.10–2.18 (d, *J* = 13.2 Hz, 2H, ring CH_2_), 2.72–2.82 (d, *J* = 13.8 Hz, 2H, ring CH_2_), 2.82–2.90 (t, *J* = 12.6 Hz, 2H, CH_2_-indole), 3.23–3.31 (d, *J* = 13.2 Hz, 2H, ring CH_2_), 4.02–4.10 (d, *J* = 13.2 Hz, 2H, ring CH_2_), 4.32–4.36 (m, 2H, CONHCH), 5.06–5.16 (d, *J* = 13.8 Hz, 2H, ring CH_2_), 6.28–6.32 (d, *J* = 6.8 Hz, 4H, indole-H), 7.06–7.34 (m, 14H, indole-H and aryl), 7.91–7.95 (d, *J* = 7.1 Hz, 2H, indole-H), 8.28 (s, 6H, NH_3_^+^), 11.12 (s, 2H, indole-NH).

^13^C NMR (CDCl_3_) (75 MHz): δ 33.1 (CH_2_-indole), 53.4 (NCHC(O)), 53.6 (ring CH_2_), 54.1 (CPh), 57.4 (ring CH_2_), 114.3 (indole CH), 120.6 (indole CH), 121.1 (indole CH), 122.4 (indole CH), 128.0 (aryl), 128.4 (indole CH), 129.2 (aryl), 129.6 (aryl), 135.9 (indole-C), 138.4 (aryl), 175.8 (NC(O)CH), 208.1 (CO).

Mass Spectrum (ES^−^): *m*/*z* 663 (M−H)^−^ (100%), 419 (10), 207 (10), 113 (20)

LRMS (ESI M + 1): 665

HRMS (ESI M + H) for C_41_H_41_N_6_O_3_: calculated 665.3235, found 665.3256

### 4.3. General Cell Procedures

Stock solutions (200 mM) of the bispidinone compounds (**BisP1**–**BisP4**) were prepared in dimethyl sulfoxide (DMSO) and stored at −20 °C. Further dilutions in media were prepared immediately prior to each experiment.

#### 4.3.1. Cell Culture

Pancreatic cancer cell lines were obtained from the American Type Culture Collection (ATCC: Manassas, VA, USA). MiaPaca-2 cells were cultured in Dulbecco’s Modified Eagle’s Medium (DMEM, Sigma-Aldrich) and supplemented with 10% foetal bovine serum (FBS, Sigma-Aldrich), 2.5% horse serum, and l-Glutamine (100 µg/mL). BxPC-3 cells were cultured in RPMI-1640 supplemented with 10% FBS and l-Glutamine (100 µg/mL). CFPAC-1 cells were cultured in Iscove’s Modified Dulbecco’s Media (IMDM, Sigma-Aldrich) supplemented with 10% FBS and l-Glutamine (100 µg/mL). All cell lines were cultured at 37 °C with 5% CO_2_.

#### 4.3.2. Cell Viability Assays

Cell viability was determined using the Dojindo Cell Counting Kit-8 (CCK-8, Dojindo Molecular Technologies, Inc., Rockville, MD, USA). Moreover, 100 µL of suspended cells were seeded into a 96 well plate at 5 × 10^3^ cells per well and allowed to adhere for 24 h. The wells were then treated with 100 µL of dissolved bispidinone derivatives at increasing concentrations (1 µM–100 µM) and a vehicle control (0.1% DMSO). Gemcitabine (50 nM) was used as a positive control. After 48 h or 72 h incubation, 10 µL of CCK-8 reagent was added to each well and further incubated at 37 °C for 2 h. The optical density of living cells was read at 450 nm using a microplate spectrophotometer (BIORAD Benchmark Plus, Bio-Rad, Hercules, CA, USA). Wells containing only media and CCK-8 reagent served as the blank. Cell viability was determined as a percentage of the control. All experiments were performed in replicates of 6. Using the mean absorbance of the vehicle control (Ac), the treated groups (A), and the blank (Ab), %viability was determined using the following equation:

(1) $$\%\;viability=\left[\left(\frac{A-Ab}{Ac-Ab}\right)\times 100\right].$$

The 50% inhibitory concentration (IC_50_) values were calculated by curve fitting the absorbance (viability) vs. log[concentration of treatment], using GraphPad Prism software Version 6.0b (GraphPad Software, Inc., San Diego, CA, USA).

#### 4.3.3. Measurement of Apoptotic Cell Morphology

MiaPaca-2 cells were seeded in 6-well plates at 1 × 10^5^ cells per well and allowed to adhere for 24 h. Cells were treated with **BisP4** at 17.5 µM and 35 µM or vehicle (0.1% DMSO) and imaged every 30 min for a total of 20 h at 20× magnification using an Axiovert 200M inverted microscope (Carl Zeiss, Inc., Oberkochen, Germany) equipped with an incubation chamber set at 37 °C/5% CO_2_. Images were viewed and analysed using AxioVision LE, Version 4.9.1.0 software (Carl Zeiss, Inc.).

#### 4.3.4. Cell Death Analysis by Fluorescent Staining 

To visually observe the effect of bispidinone treatment on the viability of pancreatic cancer cells, two different fluorescent cellular staining techniques were used. In preparation for both cell staining techniques, MiaPaca-2 cells were seeded into 12-well plates containing round coverslips, at a density of 5 × 10^4^ cells per well and allowed to adhere for 24 h at 5% CO_2_/37 °C. Cells were then treated with either 0.1% DMSO (control) or **BisP4** at concentrations of 17.5 µM, 26 µM, or 35 µM, and further incubated for 24 h before staining.

After treatment, DAPI staining was prepared by gently washing the cells with 3.7% formaldehyde in PBS solution for 5 min. Cells were then permeabilized in 1mL of permeabilization buffer (0.01 M Glycine, 0.1% Triton X-100 diluted in 3.7% formaldehyde/PBS solution), left for 5 min before aspiration, followed by another 5 min wash in 3.7% formaldehyde/PBS solution. The DAPI 4’,6-diamino-2-phenylindole stain was prepared immediately prior to use (10 µg/mL DAPI dissolved in 3.7% formaldehyde solution) and protected from light. DAPI stain (1000 µL) was added to each well and left for 5 min at room temperature, protected from light. The stain was then removed, followed by a final wash with the 3.7% formaldehyde/PBS solution. The coverslips were then mounted and viewed (20x objective) with an Axiovert 200M inverted microscope (Carl Zeiss, Inc.). Images were viewed and analyzed using AxioVision LE, Version 4.9.1.0 software.

LIVE/DEAD^®^ staining was prepared by gently washing the cells in 1000 µL of cold PBS. The coverslips were removed and placed face up on parafilm, ready for staining. The two dye reagents were combined and diluted in PBS (0.5 µM of calcein-AM and 2µM of EthD-1) immediately before use and protected from light. The coverslips were covered with 100µL of the prepared reagent, covered, and incubated for 30 min at room temperature. Following incubation, 4 µL of reagent was placed on a clean microscope slide, before carefully inverting and mounting the wet coverslip, then viewed (20× objective) with an Axiovert 200M inverted microscope (Carl Zeiss, Inc.). Images were viewed and analyzed using AxioVision LE, Version 4.9.1.0 software (Carl Zeiss, Inc.).

#### 4.3.5. Cell Death Analysis by Flow Cytometry

MiaPaca-2 cells were seeded into 6-well plates at a concentration of 1 × 10^5^ cells per well and incubated for 24 h at 37 °C with 5% CO_2_ for cells to adhere. Cells were treated for 24 h at 37 °C with 0.1% DMSO as the control and a range of **BisP4** concentrations (17.5 µM, 26 µM, or 35 µM). Following treatment, cells were assessed for apoptosis using the Muse^TM^Caspase-3/7 Kit (Merck Millipore Corp., Burlington, MA, USA) as per manufacturer’s instructions. Briefly, cells were dissociated with trypsin, resuspended in Assay Buffer and incubated for 30 min at 37 °C, protected from light in Muse™ Caspase 3/7 working reagent. After incubation, 150 µL of Muse™ Caspase 7-AAD working reagent was added, mixed thoroughly, and incubated for a further 5min at room temperature, protected from light, and analyzed with the Muse™ Cell Analyser (Merck Millipore Corp.) using Muse™ software Version 1.4.

### 4.4. Statistical Analysis

GraphPad Prism Version 6.0b software (GraphPad Software, Inc.) was used for statistical and graphical evaluations. Results are expressed as the mean ±SD of at least six replicates, unless otherwise stated. Statistical comparisons between the differences in cell viability of treatment groups compared to controls were performed using a one-way ANOVA and a post-hoc Dunnett’s Multiple Comparisons test. Statistical significance was set at *p* < 0.05.

## 5. Conclusions 

Further work is needed to elucidate the detailed molecular mechanisms of the cytotoxic effect. This will lead to potential opportunities of structural optimization of bispidinone derivative **BisP4** to improve potency and targetability. The versatility and relatively straightforward synthesis of bispidinone derivatives provides the potential for further discovery and development of bispidinone derivatives. The necessary demand for novel pancreatic cancer drug treatments and the results of this study of apoptosis-mediated cytotoxicity from in vitro treatment with a bispidinone derivative, makes this an important effort towards the development of potential novel lead compounds to target pancreatic cancer.

## Figures and Tables

**Table 1:** Structures of four bispidinone derivatives and their effect on pancreatic cancer cell viability.

Bispidinones		Significantly Reduced Cell Viability at ≤50 μM		
		MiaPaCa-2	CFPAC1	BxPC3
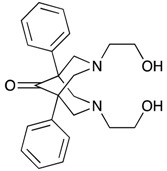	1,5-Diphenyl-3,7-bis(2-hydroxyethyl)-3,7-diazabicyclo[3.3.1]nonan-9-one (**BisP1**)	No	No	No
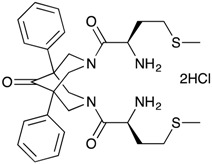	3,7-Bis-(2-(*S*)-amino-4-methylsulfanylbutyryl)-1,5-diphenyl-3,7-diazabicyclo[3.3.1]nonan-9-one dihydrochloride (**BisP2**)	No	No	No
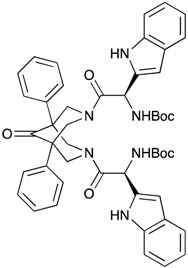	[2-{7-[2-(*S*)-*tert*-Butoxycarbonylamino-3-(1*H*-indol-3-yl)-propionyl]-9-oxo-1,5-diphenyl-3,7-diazabicyclo[3.3.1]non-3-yl}-1-(*S*)-(1*H*-indol-3-ylmethyl)-2-oxoethyl]-carbamic acid *tert*-butyl ester (**BisP3**)	ND	ND	ND
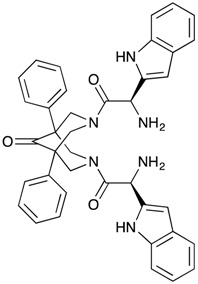	3,7-Bis-[2-(*S*)-amino-3-(1*H*-indol-3-yl)-propionyl]-1,5-diphenyl-3,7-diazabicyclo[3.3.1]nonan-9-one dihydrochloride (**BisP4**)	Yes ^a^	Yes ^a^	Yes ^b^

Cytotoxicity at 10 μM ^a^ and 30 μM ^b^. ND = No Data, compound insoluble in testing medium.

**Table 2:** Mean percentage (%) of gated events (*n* = 3, ±SD), corresponding to cells that are viable (% Live), in the early stages (% Apoptotic) and late stages of apoptosis (% Apoptotic/Dead), and dead (% Dead), following 24 h **BisP4** treatment and control.

MiaPaca-2 Treatment	% Live	% Apoptotic	% Apoptotic/ Dead	% Dead	% Total Apoptotic
**Control**	79.8 ± 0.7	5.8 ± 1.5	10.9 ± 2.0	4.0 ± 3.0	16.2 ± 3.5
**17.5 µM**	77.8 ± 5.5	4.7 ± 2.3	15.2 ± 3.7	2.3 ± 0.3	19.9 ± 5.9
**26 µM**	35.8 ± 4.1	1.2 ± 0.2	57.2 ± 4.0	5.8 ± 0.4	59.5 ± 2.5

## References

[1] Siegel R.L., Miller K.D., Jemal A. (2015). Cancer statistics, 2015. CA Cancer J. Clin..

[2] Rahib L., Smith B.D., Aizenberg R., Rosenzweig A.B., Fleshman J.M., Matrisian L.M. (2014). Projecting cancer incidence and deaths to 2030: The unexpected burden of thyroid, liver, and pancreas cancers in the United States. Cancer Res..

[3] Oberstein P.E., Olive K.P. (2013). Pancreatic cancer: Why is it so hard to treat?. Ther. Adv. Gastroenterol..

[4] Long J., Zhang Y., Yu X., Yang J., LeBrun D.G., Chen C., Yao Q., Li M. (2011). Overcoming drug resistance in pancreatic cancer. Expert Opin. Ther. Targets.

[5] Cragg G.M., Grothaus P.G., Newman D.J. (2014). New horizons for old drugs and drug leads. J. Nat. Prod..

[6] Gomtsyan A. (2012). Heterocycles in drugs and drug discovery. Chem. Heterocycl. Compd..

[7] Borsodi A., Benyhe S., Holzgrabe U., Márki Á., Nachtsheim C. (1994). Structurally novel group of ligands selective for kappa opioid receptors. Regul. Pept..

[8] Mineur Y.S., Somenzi O., Picciotto M.R. (2007). Cytisine, a partial agonist of high-affinity nicotinic acetylcholine receptors, has antidepressant-like properties in male C57BL/6J mice. Neuropharmacology.

[9] Perez E.G., Mendez-Galvez C., Cassels B.K. (2012). Cytisine: A natural product lead for the development of drugs acting at nicotinic acetylcholine receptors. Nat. Prod. Rep..

[10] Rouden J., Lasne M.C., Blanchet J., Baudoux J. (2014). (−)-Cytisine and derivatives: Synthesis, reactivity, and applications. Chem. Rev..

[11] Ruenitz P.C., Mokler C.M. (1977). Analogues of sparteine. 5. Antiarrhythmic activity of selected *N*,*N*′-disubstituted bispidines. J. Med. Chem..

[12] Galasso V., Goto K., Miyahara Y., Kovac B., Klasnic L. (2002). On the structure and spectroscopic properties of bispidine, *N*,*N*′-dimethylbispidine and a bis-bispidine macrocycle. Chem. Phys..

[13] Vatsadze S.Z., Krut’ko D.P., Zyk N.V., Zefirov N.S., Churakov A.V., Churakov A.M., Howard J.A. (1999). First ^1^H NMR observation of chair-boat conformers in bispidinone system. Molecular structure of 3,7-diisopropyl-1,5-diphenyl-3,7-diazabicyclo-[3.3.1]nonane-9-one. Mendeleev Commun..

[14] Tomassoli I., Gundisch D. (2016). Bispidine as a Privileged Scaffold. Curr. Top. Med. Chem..

[15] Haridas V., Sadanandan S., Sharma Y.K., Chinthalapalli S., Shandilya A. (2012). Bispidine as a secondary structure nucleator in peptides. Tetrahedron Lett..

[16] Haridas V., Rajgokul K.S., Sandanandan S., Agrawal T., Sharvani V., Gopalakrishna M.V.S., Bijesh M.B., Kumawat K.L., Basu A., Medigeshi G.R. (2013). Bispidine-Amino Acid conjugates act as a novel scaffold for the design of antivirals that block Japanese encephalitis virus replication. PLoS Negl. Trop. Dis..

[17] Chiavarelli S., Settimj G., Alves H. (1957). Synthesis of 1,5-diphenylbispidin-9-one and 1,5-diphenylbispidin-9-ol series. Gazz. Chim. Ital..

[18] Chiavarelli S., Toeffler F., Landi Vittory R., Mazzeo P. (1964). Synthesis of 1,5-diphenylbispidin-9-ones. VII. 1,5-difenil-3,7-dialkylbispidinones. Gazz. Chim. Ital..

[19] Jeyaraman R., Avila S. (1981). Chemistry of 3-azabicyclo[3.3.1]nonanes. Chem. Rev..

[20] Settimj G., Landi-Vittory R., Delle Monache F., Chiavarelli S. (1966). Synthesis of 1,5-diphenylbispidin-9-one and -9-ol derivatives. X. Nitration of dibenzylketone and synthesis of some 3,5-bis(nitrophenyl)-4-piperidones and 1,5-bis(nitrophenyl)-9-bispidinones. Gazz. Chim. Ital..

[21] Juran S., Walther M., Stephan H., Bergmann R., Steinbach J., Kraus W., Emmerling F., Comba P. (2009). Hexadentate bispidine derivatives as versatile bifunctional chelate agents for copper(II) radioisotopes. Bioconj. Chem..

[22] Yi M., Parthiban P., Hwang J., Zhang X., Jeong H., Park D.H., Kim D.K. (2014). Effect of a bispidinone analog on mitochondria mediated apoptosis in HeLa cells. Int. J. Oncol..

[23] Yi X., Zhang X., Jeong H., Shin Y.M., Park D.H., You S., Kim D. (2015). A novel bispidinone analog induces S-phase cell cycle arrest and apoptosis in HeLa human cervical carcinoma cells. Oncol. Rep..

[24] Black D.S.C., Deacon G.B., Rose M. (1995). Synthesis and metal complexes of symmetrically N-substituted bispidinones. Tetrahedron.

[25] Hughes J.P., Rees S., Kalindjian S.B., Philpott K.L. (2010). Principles of early drug discovery. Br. J. Pharmacol..

[26] Goldstein D., El-Maraghi R.H., Hammel P., Heinemann V., Kunzmann V., Sastre J., Scheithauer W., Siena S., Tabernero J., Teixeira L. (2015). nab-Paclitaxel plus gemcitabine for metastatic pancreatic cancer: Long-term survival from a phase III trial. J. Natl. Cancer Inst..

[27] Saif M.W., Lee Y., Kim R. (2012). Harnessing gemcitabine metabolism: A step towards personalized medicine for pancreatic cancer. Ther. Adv. Med. Oncol..

[28] Jones S., Zhang X., Parsons D.W., Lin J.C., Leary R.J., Angenendt P., Mankoo P., Carter H., Kamiyama H., Jimeno A. (2008). Core signaling pathways in human pancreatic cancers revealed by global genomic analyses. Science.

[29] Biankin A.V., Waddell N., Kassahn K.S., Gingras M.C., Muthuswamy L.B., Johns A.L., Miller D.K., Wilson P.J., Patch A.M., Wu J. (2012). Pancreatic cancer genomes reveal aberrations in axon guidance pathway genes. Nature.

[30] Waddell N., Pajic M., Patch A.M., Chang D.K., Kassahn K.S., Bailey P., Johns A.L., Miller D., Nones K., Quek K. (2015). Whole genomes redefine the mutational landscape of pancreatic cancer. Nature.

[31] Shendage D., Frohlich R., Haufe G. (2004). Highly efficient stereoconservative amidation and deamidation of α-amino acids. Org. Lett..

[32] Hanahan D., Weinberg R.A. (2011). Hallmarks of Cancer: The Next Generation. Cell.

[33] Brunelle J.K., Zhang B. (2010). Apoptosis assays for quantifying the bioactivity of anticancer drug products. Drug Resist. Updat..

[34] Portugal J., Bataller M., Mansilla S. (2009). Cell death pathways in response to antitumo therapy. Tumori.

